# Effects of helmet nonuse and seating position on patterns and severity of injuries in child motorcycle passengers

**DOI:** 10.1186/s12889-019-7434-5

**Published:** 2019-08-08

**Authors:** Hsiu-Ping Fan, Wen-Ta Chiu, Mau-Roung Lin

**Affiliations:** 1 Institute of Injury Prevention and Control, College of Public Health, Taipei Medical University, 250 Wu-Hsing St, Taipei, 110 Taiwan; 2 Division of Emergency Medicine, Department of Emergency and Critical Care Medicine, Fu Jen Catholic University Hospital, Fu Jen Catholic University, 69 Guizi Road, Taishan District, New Taipei City, 243 Taiwan; 3 School of Medicine, Fu-Jen Catholic University, 510 Zhongzheng Rd., Xinzhuang Dist., New Taipei City, 242 Taiwan; 4 Graduate Institute of Injury Prevention and Control, Taipei Medical University, 250 Wu-Hsing Street, Taipei, 11031 Taiwan

**Keywords:** Child, Motorcycle passenger, Helmet use, Risky behavior, Injury severity

## Abstract

**Background:**

A prospective study was conducted to investigate the effects of helmet nonuse and seating position on patterns and severity of motorcycle injuries among child passengers in Taiwan.

**Methods:**

In total, 305 child passengers aged ≤14 years who visited the emergency departments of three teaching hospitals following a motorcycle crash were recruited. Children’s injury data were collected from medical records, and their riding behaviors along with operators’ demographics were sourced from telephone interviews. Parental responses over the telephone about children’s riding behaviors were checked by roadside observations.

**Results:**

Results of the multivariable logistic regression analysis revealed that compared to child passengers aged ≥7 years, those aged ≤3 (odds ratio (OR), 2.88; 95% confidence interval (CI), 1.37~6.06) and 4~6 years (OR, 2.93; 95% CI, 1.50~5.70) were significantly more likely to have sustained a head/face injury, while those aged 4~6 years (OR, 2.76; 95% CI, 1.01~7.55) were significantly more likely to have sustained a severe injury. Compared to child passengers who were wearing a full-coverage helmet, those who were not wearing a helmet were significantly more likely to have sustained a head/face injury (OR, 3.12; 95% CI, 1.02~9.52) and a severe injury (OR, 3.02; 95% CI, 1.19~7.62). Children seated in front of the operator were significantly more likely to have experienced a head/face injury (OR, 2.22; 95% CI, 1.25~3.94) than those seated behind the operator. For each increment in the riding speed of 1 km/h, the odds of a severe injury to child passengers increased by 5% (OR, 1.05; 95% CI, 1.01~1.09).

**Conclusions:**

For the safety of child motorcycle passengers, laws on a minimum age restriction, helmet use, an adequate seating position, and riding speed need to be enacted and comprehensively enforced.

## Background

Children on motorcycles are one of the most vulnerable populations on the road [1], and pediatric motorcycle injuries have emerged as an important public health problem and have attracted worldwide attention [2–10]. In the US and other developed countries where motorcycles are usually used for recreation, an increasing number of off-road motorcycle injuries to children have been reported [2–5]. On the other hand, a great number of pediatric motorcycle injuries on roads come from the Southeast Asian and Western Pacific regions where motorcycles are widely adopted and are a commonly used mode of transport [11]. For example, in Taiwan, motorcycles account for two-thirds of all registered motor vehicles and result in approximately half of road traffic deaths [12].

Helmet use was found to reduce motorcycle fatalities by 42% and head injuries by 69% [13], and universal helmet laws can further increase helmet usage, reduce motorcycle fatalities and injuries, and lower societal costs [14]. Nonetheless, even in countries with a universal helmet law, the prevalence of helmet use in child passengers is approximately one-third to one-fifth of helmet use in adult passengers [15–17]. Several other risky behaviors are also prevalent in child passengers on motorcycles, such as multiple passengers on a motorcycle and sitting on the petrol tank of a standard motorcycle or standing/sitting in the front area of a scooter [1, 17, 18].

While motorcycles are the most commonly used vehicle for short-range transportation in Taiwan and other countries in Southeast Asia, the majority of these motorcycles are scooters that can easily carry young child passengers in the front area. Accordingly, we conducted this prospective study to examine the effects of helmet nonuse, seating position, and other riding behaviors on patterns and the severity of motorcycle injuries in child passengers.

## Methods

A 19-month prospective study was conducted to investigate effects of helmet nonuse and seating position on patterns and severity of motorcycle injuries among child passengers in Taiwan.

### Study settings and participants

During the period of July 2011 to January 2013, all motorcycle passengers aged < 14 years who visited the emergency department (ED) of three teaching hospitals immediately after a motorcycle crash were eligible for the study. The three participating hospitals (Changhua Christian Hospital, Show Chwan Memorial Hospital, and Chang Bing Show Chwan Memorial Hospital) account for about 80% of ED visits in Changhua County, west-central Taiwan.

After stabilization of the injury conditions for hospitalization or at the time of preparing for discharge from the ED, parents or grandparents of eligible child motorcycle passengers were invited to participate in the study. If the parents or grandparents agreed, their contact information was obtained, and researchers conducted a 20-min interview with the parents over the telephone within approximately 2 weeks after the ED visit. On the contrary, child passengers were excluded if they were the motorcycle operator, if the motorcycle used had more than two wheels or training wheels, or if the motorcycle was powered by anything other than gasoline (e.g., an electric scooter) in that only very few people used non-two-wheeled or electric motorcycles during the study period, and mechanisms of crashes for these non-typical motorcycles would differ.

This research was approved by the institutional review boards of Taipei Medical University and the three participating hospitals, and written informed consent was obtained from each participating child’s parent or legal guardian in this study.

### Data collection

Data on injury characteristics and riding behaviors of child passengers were collected from medical records and parents’ telephone interviews. Injury characteristics consisted of the body regions injured, injury severity, and disposition after ED treatment (admission to an intensive care unit (ICU), admission to a ward, or discharged home). Riding behaviors consisted of the helmet status, seating position, total number of passengers on the motorcycle, and the riding speed immediately prior to the crash. In addition, information on the parental driver’s gender, age, and educational level was also collected.

Injury severity was assessed by the Abbreviated Injury Score (AIS) [19] and Injury Severity Score (ISS) [20]. In the AIS and ISS measures, anatomical body regions are classified into six parts: the head, face, chest, abdomen, extremities, and external. The AIS assigns each body region a severity score ranging from 1 (minor) to 6 (maximum), with a higher score indicating a more-severe injury. For an individual who sustains injuries involving multiple body regions, the ISS calculates the sum of the squares of the highest AIS scores for the three different most severely injured body regions to estimate the overall injury severity. Here, ISS scores of ≥5 were defined as a severe injury.

Wearing a standard helmet, such as a full-coverage helmet (including full-faced which covers the entire haired region of the head and both ears and with a chin bar, and open-faced which covers the cheeks but without a chin bar) or a half-coverage helmet (covering the head above the ears but exposing both ears and the lower part of the occiput), was defined as being helmeted. Conversely, not wearing a helmet or wearing a nonstandard helmet (an industrial helmet, a bicycle helmet, or an in-line skating helmet) was defined as being unhelmeted. A front riding position was considered when a child passenger stood/sat in front of the operator, while a back position was when seated behind the operator. Carrying multiple passengers was considered when there were two or more passengers on a motorcycle.

### Validation of riding behaviors

We also collected a population-based sample of child motorcycle passengers to check parental responses to the telephone interviews on children’s riding behaviors in the ED study sample. During five Saturdays in June 2012, researchers observed people who were riding motorcycles with child passengers at the motorcycle parking lot of the only shopping mall in Changhua City in which the total number of passengers on the motorcycle and the helmet type and seating position of all child passengers were observed and recorded. Immediately after the observation, the motorcycle operator was invited to participate in the study; if they agreed, the operator was further interviewed to collect information on the age and sex of child passengers and the highest speed of the riding journey. For comparison with the ED sample, only one child passenger from each motorcycle was selected using a random number table in order to avoid a sample with dependent observations. The observed data were excluded when the age of a child passenger exceeded 14 years.

### Statistical analysis

Using Pearson’s Chi-squared or Fisher’s exact test for categorical variables and Student’s *t*-test for continuous variables, the distributions of age, sex, injury severity, and disposition after the ED visit between respondents and non-respondents were compared to examine potential sampling bias in the study.

Riding behaviors and demographic characteristics of child passengers and parental operators between groups with respect to the presence of injury to each body region (AIS ≥ 1) and severe injury (ISS ≥ 5) were compared using Pearson’s Chi-squared or Fisher’s exact test for categorical variables and Student’s *t*-test for continuous variables. A multivariable logistic regression model was applied to investigate whether the odds of having an injury to each body region and a severe injury independently differed among groups in terms of riding behaviors and demographics of child passengers and parental operators, after adjusting for potential confounders. While the level of significance was set to 0.05, variables with a *p* value of < 0.2 in the preliminary analysis were included in the initial multivariable analysis to minimize type II errors in variable selection and biased inferences. Because four riding behaviors, including helmet type, seating position, multiple passengers, and riding speed, were the focus of this study, they were necessarily included in the final multivariable analysis.

Finally, demographics and riding behaviors of child passengers and parental operators from the ED sample were checked by the parental responses from the parking lot sample. All analyses were performed using the Statistical Analysis Software (SAS) package (vers. 9.4 for Windows; SAS Institute, Cary, NC, USA).

## Results

During the 19-month study period, 399 child motorcycle passengers were identified in the EDs of the three study hospitals, among which 305 (76.3%) participated in the study. Compared to respondents, non-respondents were significantly more likely to have a head/face injury (*p* = 0.004) and a severe injury (*p* < 0.001) in all body regions but the external.

Distributions of injured body regions, injury severity, and disposition after the ED visit among the 305 child passengers are shown in Table 1. Of the six anatomical body regions, the extremities were the most frequently injured (occurring in 81.0% of the children), followed by the face (42.3%), head (39.7%), abdomen (11.5%), and chest (5.9%). Of the 305 child passengers, 158 (51.8%) sustained multiple injuries; 28 (9.2%) had a severe injury with an ISS of ≥5; 34 (11.1%) were admitted to an ICU or ward; and 271 (88.9%) were discharged.

**Table 1:** Distributions of injured body regions, injury severity, and disposition after an emergency department (ED) visit among 305 child motorcycle passengers

Characteristic	Child passengers (*N* = 305)	
	No.	(%)
Injured body region		
Head	121	(39.7)
Face	129	(42.3)
Chest	18	(5.9)
Abdomen	35	(11.5)
Extremity	247	(81.0)
External	2	(0.7)
Multiple (≥ 2 body regions)	158	(51.8)
Abbreviated Injury Scale (AIS)		
AIS-Head		
0	184	(60.3)
1	110	(36.1)
2	5	(1.7)
3	1	(0.3)
4	4	(1.3)
5	1	(0.3)
AIS-Face		
0	176	(57.7)
1	126	(41.3)
2	3	(1.0)
AIS-Chest		
0	287	(94.1)
1	17	(5.6)
3	1	(0.3)
AIS-Abdomen		
0	270	(88.5)
1	28	(9.2)
2	6	(2.0)
3	1	(0.3)
AIS-Extremities		
0	58	(19.0)
1	215	(70.5)
2	27	(8.9)
3	5	(1.6)
AIS-External		
0	303	(99.3)
1	2	(0.7)
Injury Severity Score (ISS)		
Mild (ISS ≤ 4)	277	(89.8)
Severe (ISS ≥ 5)	28	(9.2)
Disposition after ED visit		
Admitted to an intensive care unit	8	(2.6)
Admitted to a ward	26	(8.5)
Discharged	271	(88.9)

Demographics and riding behaviors of child passengers and parental operators with respect to an injury to a body region (AIS ≥ 1) and a severe injury (ISS ≥ 5) are summarized in Table 2. Child passengers who were younger, not wearing a helmet, and seated in front of the operator and those whose parents were aged ≤35 and ≥ 51 years and had attained a senior high educational level or less were significantly more likely to have sustained a head/face injury, compared to their counterparts. In contrast, child passengers who were older and seated behind the operator were significantly more likely to have sustained an extremity injury compared to their counterparts. No significant differences in riding behaviors of child passengers or demographic characteristics of the operator between child passengers with a chest/abdomen injury and those without were found. Child passengers who were younger, not wearing a helmet, seated in front of the operator, and traveling at higher speeds were significantly or marginally more likely to have sustained a severe injury.

**Table 2:** Demographics and riding behaviors of 305 child passengers and adult operators with respect to injured body regions and injury severity

Characteristic	Head/face injury					Chest/abdomen injury					Extremity injury					Severe injury				
	Absence (*N* = 136)		Presence (*N* = 169)		*p* value	Absence (*N* = 259)		Presence (*N* = 46)		*p* value	Absence (*N* = 58)		Presence (*N* = 247)		*p* value	ISS < 5 (*N* = 277)		ISS ≥ 5 (*N* = 28)		*p* value
	No.	(%)	No.	(%)		No.	(%)	No.	(%)		No.	(%)	No.	(%)		No.	(%)	No.	(%)	
Child passenger																				
Age (years)																				
≤ 3	19	(14.0)	58	(34.3)	< 0.001	70	(27.0)	7	(15.2)	0.223	29	(50.0)	48	(19.4)	< 0.001	71	(25.6)	6	(21.4)	0.034
4~6	21	(15.4)	50	(29.6)		58	(22.4)	13	(28.3)		17	(29.3)	54	(21.9)		59	(21.3)	12	(42.9)	
≥ 7	96	(70.6)	61	(36.1)		131	(50.6)	26	(56.5)		12	(20.7)	145	(58.7)		147	(53.1)	10	(35.7)	
Boy/girl	76	(55.9)	85	(50.3)	0.331	133	(51.4)	28	(60.9)	0.233	29	(50.0)	132	(53.4)	0.637	146	(52.7)	15	(53.6)	0.931
Helmet type																				
Unhelmeted	34	(25.6)	94	(57.0)	< 0.001	108	(42.5)	20	(45.5)	0.839	32	(55.2)	96	(40.0)	0.111	109	(40.2)	19	(70.4)	0.008
Half-coverage	87	(65.4)	64	(38.8)		129	(50.8)	22	(50.0)		23	(39.6)	128	(53.3)		143	(52.8)	8	(29.6)	
Full-coverage	12	(9.0)	7	(4.2)		17	(6.7)	2	(4.5)		3	(5.2)	16	(6.7)		19	(7.0)	0	(0.0)	
Being seated in front of the operator	44	(32.6)	111	(65.7)	< 0.001	129	(50.0)	26	(56.5)	0.415	44	(75.9)	111	(45.1)	< 0.001	136	(49.3)	19	(67.9)	0.061
Having multiple passengers	52	(38.5)	73	(43.2)	0.410	107	(41.5)	18	(39.1)	0.766	24	(41.4)	101	(41.1)	0.964	111	(40.2)	14	(50.0)	0.316
Riding speed (mean ± SD, km/h)	27.9	±13.5	30.3	±13.5	0.146	29.0	±13.0	30.4	±16.7	0.566	26.8	±11.6	29.8	±13.9	0.161	28.5	±13.3	36.5	±14.1	0.007
Adult operator																				
Age (years)																				
≤ 35	41	(36.0)	61	(42.6)	0.023	84	(38.5)	18	(46.2)	0.302	24	(51.1)	78	(37.1)	0.090	93	(39.7)	9	(39.1)	0.873
36~50	55	(48.2)	46	(32.2)		90	(41.3)	11	(28.2)		12	(25.5)	89	(42.4)		91	(38.9)	10	(43.5)	
≥ 51	18	(17.8)	36	(25.2)		44	(20.2)	10	(25.6)		11	(23.4)	43	(20.5)		50	(21.4)	4	(17.4)	
Male/female	39	(28.7)	52	(30.8)	0.691	72	(27.8)	19	(41.3)	0.065	14	(24.1)	77	(31.2)	0.292	83	(30.0)	8	(28.6)	0.878
Educational level																				
Elementary or below	16	(16.0)	26	(19.4)	0.011	36	(17.8)	6	(18.7)	0.985	5	(11.4)	37	(19.5)	0.182	38	(18.1)	4	(16.7)	0.395
High school	48	(48.0)	83	(61.9)		113	(55.9)	18	(56.3)		30	(68.2)	101	(53.1)		120	(57.1)	11	(45.8)	
College and above	36	(36.0)	25	(18.7)		53	(26.2)	8	(25.0)		9	(20.4)	52	(27.4)		52	(24.8)	9	(37.5)	

I*SS* Injury Severity Score

Results of the multivariable logistic regression analysis for an injury to the head/face, chest/abdomen, and extremity, as well as a severe injury among child passengers are shown in Table 3. After adjusting for other variables, compared to child passengers aged ≥7 years, those aged ≤3 (odds ratio (OR), 2.88; 95% confidence interval (CI), 1.37~6.06) and 4~6 years (OR, 2.93; 95% CI, 1.50~5.70) were significantly more likely to have sustained a head/face injury, while those 4~6 years old (OR, 2.76; 95% CI, 1.01~7.55) were significantly more likely to have sustained a severe injury. Furthermore, unhelmeted child passengers were significantly more likely to have sustained a head/face injury (OR, 3.12; 95% CI, 1.02~9.52), compared to those who were wearing a full-coverage helmet, and they were significantly more likely to have sustained a severe injury (OR, 3.02; 95% CI, 1.19~7.62), compared to those who were wearing a half-coverage helmet. Child passengers seated in front of the operator were significantly more likely to have experienced a head/face injury (OR, 2.22; 95% CI, 1.25~3.94) than those behind the operator. With each increment in riding speed of 1 km/h, the odds of sustaining a severe injury to child passengers increased by 5% (OR, 1.05; 95% CI, 1.01~1.09).

**Table 3:** Results of the multivariable logistic regression analysis for various injured body regions and severe injuries among child motorcycle passengers

Characteristic	Head/face injury		Chest/abdomen injury		Extremity injury		Severe injury	
	OR	(95% CI)	OR	(95% CI)	OR	(95% CI)	OR	(95% CI)
Child passenger								
Age (years)								
≤ 3	2.88	(1.37, 6.06)		NA	0.21	(0.09, 0.50)	0.80	(0.24, 2.68)
4~6	2.93	(1.50, 5.70)		NA	0.36	(0.15, 0.84)	2.76	(1.01, 7.55)
≥ 7	1.00			NA	1.00		1.00	
Helmet type								
Unhelmeted	3.12	(1.02, 9.52)	1.47	(0.31, 7.00)	0.81	(0.20, 3.32)	3.02	(1.19, 7.62)
Half-coverage	1.25	(0.43, 3.65)	1.38	(0.30, 6.47)	0.89	(0.22, 3.64)	1.00	
Full-coverage	1.00		1.00		1.00			NA*
Being seated in front of the operator	2.22	(1.25, 3.94)	1.29	(0.67, 2.49)	0.54	(0.25, 1.15)	1.73	(0.67, 4.43)
Having multiple passengers	1.04	(0.61, 1.79)	0.89	(0.46, 1.72)	0.93	(0.49, 1.77)	1.20	(0.52, 2.79)
Riding speed (mean ± SD, km/h)	1.02	(0.99, 1.04)	1.01	(0.98, 1.03)	1.01	(0.99, 1.04)	1.05	(1.01, 1.09)
Adult operator								
Educational level								
Elementary or below	2.04	(0.80, 5.23)		NA		NA		NA
High school	2.14	(1.05, 4.35)		NA		NA		NA
College and above	1.00			NA		NA		NA

*CI* Confidence interval, *NA* Not applicable, *OR* odds ratio

* No child passengers with a full-coverage helmet had a severe injury

Comparisons of demographics and riding behaviors between 305 child passengers in the ED and 193 child passengers in the parking lot are shown in Table 4. Among 305 injured child passengers, 52.8% were boys; 48.5% were of preschool age; 42.9% were unhelmeted; 51.0% were seated in front of the operator; and 41.1% were on a motorcycle with multiple passengers. The mean of riding speeds prior to the crash was 29.2 km/h. Compared to child passengers in the parking lot, those at the ED were older and were riding at lower speeds, while no significant differences in the helmet status, helmet type, seating position, or multiple passengers between the two groups were detected. In addition, compared to parental operators in the parking lot, those at the ED were older and had lower educational levels.

**Table 4:** Distributions of demographics and riding behaviors between 305 child passengers with motorcycle injuries at the emergency department and 193 child passengers in a parking lot of a shopping mall

Characteristic	Child passengers in the ED (*N* = 305)		Child passengers in a parking lot (*N* = 193)		*p* value
	No.	(%)	No.	(%)	
Child passenger					
Age (mean ± SD, years)	7.1	±3.5	6.6	±2.9	
≤ 3	77	(25.2)	42	(21.8)	0.021
4~6	71	(23.3)	67	(34.7)	
≥ 7	157	(51.5)	84	(43.5)	
Sex					
Boy	161	(52.8)	99	(53.5)	0.876
Girl	144	(47.2)	86	(46.5)	
Helmet type					
Unhelmeted	128	(42.9)	77	(40.3)	0.653
Half-coverage	151	(50.7)	98	(51.3)	
Full-coverage	19	(11.4)	16	(8.4)	
Seating position					
In front of the operator	155	(51.0)	109	(56.8)	0.209
Behind the operator	149	(49.0)	83	(43.2)	
Multiple passengers					
Yes	125	(41.1)	91	(47.4)	0.170
No	179	(58.9)	101	(52.6)	
Riding speed (mean ± SD, km/h)	29.2	±13.6	42.0	±12.7	< 0.001
Adult operator					
Age (years)	40.7	±13.1	36.5	±5.7	< 0.001
≤ 35	102	(39.7)	77	(40.1)	< 0.001
36~50	101	(39.3)	113	(58.9)	
≥ 51	54	(21.0)	2	(1.0)	
Sex					
Male	91	(29.8)	63	(32.6)	0.509
Female	214	(70.2)	130	(67.4)	
Educational level					
Elementary or below	42	(17.9)	5	(2.6)	< 0.001
High school	131	(56.0)	90	(46.6)	
College and above	61	(26.1)	98	(50.8)	

## Discussion

Helmet use was significantly associated with a lower risk of head/face injuries while it was not associated with a higher or lower risk of chest/abdomen or extremity injuries in child motorcycle passengers, indicating a causal effect of helmets in reducing head/face injuries. Helmet use also prevented the occurrence of a severe injury (ISS ≥ 9), in which more than half (58.3%) had a more-severe head injury with an AIS of ≥2. It is common for child motorcycle passengers to not wear a helmet, even in countries with a mandatory helmet use law [21]. Similar to previous studies conducted in both developing and developed countries (20%~ 35%) [15–17], 42.9% of the study sample did not wear a helmet. Furthermore, the effect of the half-coverage helmet in reducing head injuries in child passengers somewhat differed from previous results for adult riders. For adult motorcycle riders, head protection by half-coverage helmets was less effective (OR, 2.06; 95% CI, 1.34~3.17) than the full-coverage ones [22]. Further studies are needed to validate this result, although nonstandard or improper use of helmets by child passengers might play a role in their ineffectiveness.

Preschool-aged passengers were more likely to sustain a head/face injury than were school-aged ones. One possible explanation is the relative larger head-to-body ratio of preschool-aged children, because the human head-to-body ratio is about one-quarter in a newborn and gradually decreases until adulthood. Additionally, given that preschoolers are shorter, their head during a crash might be more likely to contact the handlebars of motorcycle, particularly for those standing or sitting in the frontal area. On the other hand, weaker muscle control and poorer self-protecting ability may also lead younger children to have increased risks of head and severe injuries. Relative to younger child passengers, older child passengers may have developed better self-protecting responses and might use their extremities to shield their head, resulting in a higher risk of extremity injuries during a motorcycle crash. Currently, 46 countries have enacted laws to specify a minimum age for child passengers on motorcycles, ranging 3~14 years [11]. Few countries require that child motorcycle passengers be tall enough for their feet to reach the foot pegs of the motorcycle [23], while many countries, such as Taiwan, still have no minimum age and only a few states in the US have a minimum legal age for motorcycle passengers. However, empirical evidence of age restrictions or anthropometric limits reducing the risk of motorcycle injuries being effective is needed.

Although it is well-known that young child occupants in the front seat of passenger cars have an increased risk of mortality and serious injury than those in the rear seat [24, 25], this study provides the first evidence to support that child motorcycle passengers standing/sitting in front of the operator are more likely to sustain head/face injuries than those behind the operator. In fact, it is a common phenomenon in Taiwan and Southeast Asian countries that a motorcycle carries multiple passengers, with a child or even two children in front of the operator [1], partly because the majority of motorcycle designs in these countries are scooter-like. In Taiwan, a young child passenger usually stands on the front footrest between the legs of the adult operator and an older child passenger sits on the seat behind the operator; therefore, the mean age of child passengers in the front was significant younger than that of child passengers in the rear (5.2 vs. 9.1 years). A decelerating force during a crash would cause the head/face of the child passenger in front of the operator to hit the handlebar, while the head/face of the child passenger behind the operator would be protected by the operator’s back.

Riding speeds were associated with the occurrence of severe injuries in child passengers. Higher speeds may increase the risk of crash involvement because they result in shorter response times, longer stopping distances, and greater difficulty controlling the vehicle after a braking event [11]. Importantly, the risk of death and serious injury among motorcycle riders is also greater at higher speeds [26], in that the amount of kinetic energy exponentially increases with a constant acceleration of the speed at impact in a crash. Moreover, child passengers are vulnerable to being thrown off the motorcycle during a high-speed motorcycle crash, resulting in an injury and even a severe injury [23].

A major difference between adult or adolescent studies and child studies of motorcycle injuries is that the latter need to consider characteristics of both the child passengers and adult operators (usually parents or grandparents). In this study, lower education levels of motorcycle operators were associated with the occurrence of head/face injuries to child passengers. Similarly, a Swedish population-based cohort study also reported a low parental socioeconomic status (manual laborers) being associated with at an increased risk of child motorcycle injuries [27]. Since educational attainment is associated with safety perception and compliance with safety procedures [28], perhaps, parental motorcyclists with lower education levels tended to wear helmets improperly, thereby increasing their risk of head/face injuries during a crash. Nevertheless, it should be noted that the relationship between children’s injury risk and parental socioeconomic status or educational level could be confounded by family financial stress, parental personality traits, and the community environment of the residence.

The original purpose of the parking lot sample was to check the data on riding behaviors in responses from the study sample collected from the ED of the three hospitals. However, some differences in these characteristics between the two samples may have also reflected differences of motorcycle riding between weekdays and the weekend, since the data from the parking lot sample were collected on Saturdays only. It is reasonable that parents are more available to carry children to outdoor activities during weekends, while grandparents are more likely to help parents carry children to school during weekdays. Furthermore, in Taiwan, older people often have lower educational levels and lower riding speeds than younger people.

There are several limitations to this study. First, child passengers who did not visit the ED after a motorcycle crash, such as those who died on the scene, had a minor injury, or did not have an injury, were not included in this study, and their characteristics and injury patterns, as well as parental characteristics, may have differed from those of study participants. Therefore, generalizing these results to all child passengers should be done with caution. For instance, child passengers who sustained a head/face injury and a severe injury tended not to be included in the study, and the protective effect of helmet use might have been underestimated. Second, potential confounding factors related to the vehicle (e.g., size, design, and stroke volume) and environment (e.g., community environment and weather) were not assessed or controlled for in the study. Third, measurement errors of the self-reported data might be large, and social desirability effects might exist in the responses to the items of helmet use and riding speed. Finally, there was a lack of information on whether the helmets were used properly (e.g., helmet fit and fixation status), and these factors may have skewed the effectiveness of helmet use. In practice, it is difficult to determine the fitness and fixation of a helmet because very few riders bring the child’s helmet to the ED.

## Conclusions

Preschool age, seating position in front of operator, and higher riding speeds may increase risks of head/face injury or severe injury to child motorcycle passengers. While more research is needed to confirm our result, we believe that enactment of laws on a minimum age restriction, helmet use, an adequate seating position, and low riding speeds can benefit for the safety of child motorcycle passengers*.*

## Data Availability

Data and material are available in the lab of Mau-Roung Lin, Graduate Institute of Injury Prevention and Control, Taipei Medical University.

## Abbreviations

AIS: Abbreviated Injury Score
ED: Emergency department
ICU: Intensive care unit
ISS: Injury Severity Score
